# Potential applications of aptamers in veterinary science

**DOI:** 10.1186/s13567-021-00948-4

**Published:** 2021-06-02

**Authors:** Solène Niederlender, Jean-Jacques Fontaine, Grégory Karadjian

**Affiliations:** 1 École Nationale Vétérinaire d’Alfort, Université Paris-Est Sup, 7 avenue du Général de Gaulle, 94700 Maisons-Alfort, France; 2 UMR BIPAR 956, ANSES, INRAE, École Nationale Vétérinaire d’Alfort, Université Paris-Est Sup, 7 avenue du Général de Gaulle, 94700 Maisons-Alfort, France; 3 UMR BIPAR, Laboratoire de Santé Animale, ANSES, INRAE, École Nationale Vétérinaire d’Alfort, Université Paris-Est Sup, 94700 Maisons-Alfort, France

**Keywords:** Aptamer, Application, Food and environmental safety, Diagnostics, Therapeutics, Veterinary sciences

## Abstract

Aptamers are small nucleic acids that fold in a three-dimensional conformation allowing them to bind specifically to a target. This target can be an organic molecule, free or carried in cells or tissues, or inorganic components, such as metal ions. Analogous to monoclonal antibodies, aptamers however have certain advantages over the latter: e.g., high specificity for their target, no to low immunogenicity and easy in vitro selection. Since their discovery more than 30 years ago, aptamers have led to various applications, although mainly restricted to basic research. This work reviews the applications of aptamers in veterinary science to date. First, we present aptamers, how they are selected and their properties, then we give examples of applications in food and environmental safety, as well as in diagnosis and medical treatment in the field of veterinary medicine. Because examples of applications in veterinary medicine are scarce, we explore the potential avenues for future applications based on discoveries made in human medicine. Aptamers may offer new possibilities for veterinarians to diagnose certain diseases—particularly infectious diseases—more rapidly or “at the patient’s bedside”. All the examples highlight the growing interest in aptamers and the premises of a potential market. Aptamers may benefit animals as well as their owners, breeders and even public health in a “One Health” approach.

## Introduction

In the most restrictive sense, aptamers are single-stranded nucleic acid sequences that fold in a complex three-dimensional shape, giving them the ability to bind strongly to a specific target [1]. There are DNA (deoxyribonucleic acid) or RNA (ribonucleic acid) aptamers. Peptide aptamers also exist, but they will not be explored here because they do not meet the strict definition of an aptamer as a molecule made up of nucleic acids. The term aptamer is a combination of the Latin word “aptus”, which means “adapted to”, and the Greek word “meros” meaning “part”, which is used to indicate the smallest unit of a repeating structure [2]. The interaction between the aptamer and its target can be described by a dissociation constant, or K_d_, whose value corresponds to the concentration of aptamers at which 50% of the targets are bound. The lower a K_d_ value is, the higher the affinity between an aptamer and its target is. Aptamers show high affinity for their targets, with K_d_ values varying from the micromolar (10^–6^ M) to the picomolar (10^–12^ M) range [3]. They also have the ability to bind specifically to their target, meaning they can discriminate structurally similar ligands [2]. Their specificity can be quantitatively measured as the ratio of K_d_ for the cognate target relative to K_d_ for a non-cognate target. Nonetheless, specificity is rarely quantified in studies, which often focus solely on the affinity of the aptamers and therefore their sensitivity. Aptamers have been studied for almost 30 years, but their large-scale commercialization and use are still in their infancy. Certain limitations, such as low chemical diversity, susceptibility to nucleases and rapid clearance within an organism, are obstacles to their use. In recent years, the number of articles on aptamers has grown exponentially and many solutions have been found to work around these limitations.

Although largely confined to basic research, the potential applications of aptamers are numerous. These applications include the detection of a toxic molecule or a molecule of interest in the environment or in food, or the detection of a pathogen. They are versatile molecules with great potential to develop rapid, portable and potentially re-usable detection tools. The diagnostic possibilities in human and veterinary medicine are numerous, ranging from infectious diseases to oncology, and can be combined with medical imaging techniques. Therapeutic applications are also envisaged. Although there is currently only one aptamer on the drug market, many more are in clinical trials and may enter the drug market in coming years. The applications in veterinary medicine are less advanced than in human medicine, but the latter can be adapted and transposed to serve animal health, as well as in a “One Health” approach. In particular, in the field of infectious diseases, aptamers have the highest potential for transposition from human medicine to veterinary medicine and the need for diagnostic tools is increasingly important.

The goal of this review is to establish an inventory of current knowledge and applications involving aptamers in veterinary fields, i.e., food safety, environmental safety and veterinary medicine. It is essential first to define and briefly present aptamers to better understand their potential applications. We frequently compare them with monoclonal antibodies, which constitute their benchmark, but also their competitor in most fields. The final goal is to illustrate the growing potential of aptamers as diagnostic tools and therapeutic drugs in veterinary medicine.

## The aptamer selection process and aptamer properties

### Selection process

**Figure 1:**
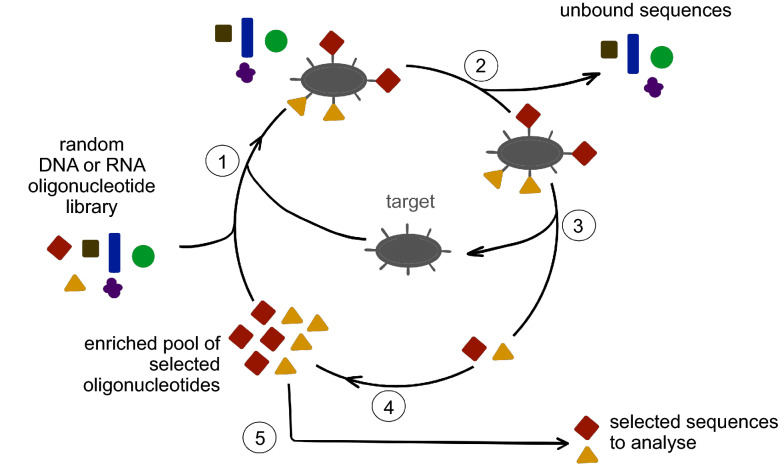
**Schematic of the SELEX process.** Diagram representing the different steps of the SELEX process to obtain aptamers. (1) Incubation with the target. (2) Partitioning. (3) Elution/separation. (4) Amplification with PCR or RT-PCR. (5) After the last SELEX round, cloning of the selected aptamer pool.

Aptamers are selected from combinatorial DNA or RNA libraries using the systematic evolution of ligands by exponential enrichment (SELEX) technology, first described in 1990 by Tuerk and Gold [4]. SELEX consists of cycles of incubation, separation and amplification [5]. The different steps of a SELEX process are shown in Figure 1.

Any SELEX process begins with a library of 10^13^ to 10^15^ synthetic DNA oligonucleotides. These oligonucleotides are made of a random central sequence of predetermined length, varying from 20 to 80 nucleotides. This random sequence is flanked on both sides by a specific fixed sequence of 18 to 21 nucleotides, which will be used for designing primers for polymerase chain reaction (PCR). To select RNA aptamers, this DNA library must first be transcribed into an RNA library using a T7 RNA polymerase [6]. During the first step, the library is incubated with the target molecule. The oligonucleotides that do not bind are filtered and removed from the solution through a second step called partitioning. Those that have bound with the target are considered “aptamers” and are separated from their target in following elution step. Following this separation, their sequences are amplified by PCR or reverse transcription PCR (RT-PCR), depending on whether they are DNA or RNA sequences. The new pool of oligonucleotides then undergoes a new cycle of SELEX. Iterative rounds are carried out until only the oligonucleotides with the most affinity and specificity for the target remain. It usually takes 6 to 20 rounds to identify and isolate specific aptamers. The products of the final PCR are separated, cloned and ideally sequenced and analyzed [5].

Since its creation in 1990, the SELEX process has been adopted and many variations have been developed to circumvent certain disadvantages of conventional SELEX. Each variation has its own objectives, e.g., to optimize the initial technique to save time and money or to obtain higher affinity and specificity. The widely used SELEX techniques are summarized in Table 1 with their objectives and their advantages over the conventional SELEX process. The techniques regularly encountered in veterinary research are cell-SELEX, capillary electrophoresis SELEX (CE-SELEX) and Mag-SELEX, possibly combined with counter and negative SELEX [5–11]. Post-SELEX modifications are also possible and often carried out.

**Table 1:** **Modified SELEX techniques and their advantages**

Modified SELEX techniques	Principle	Advantages	References
Negative SELEX	Removing the nucleic acid sequences that bind to the matrix, on which the targets are attached	Increased affinity of the selected aptamers	[7]
Counter SELEX	Removing non-specific nucleic acid sequences from the target by performing negative selection on a molecule that is structurally close to the target	More specific aptamers	[7]
CE-SELEX	Selection on capillary electrophoresis, therefore more efficient separation of linked sequences from unbound sequences, in solution	Use of non-immobilized targets, fewer cycles required (therefore less expensive because less sample and less solvent required)	[5, 7, 8]
MonoLEX	A single selection step by affinity chromatography	Only one step, therefore much faster	[9]
Mag-SELEX	With oligonucleotides and/or targets attached to metal beads, separation of bound and unbound sequences by a magnetic field	Use of smaller targets, easier and faster separation step	[5, 7]
Microfluidic SELEX	Use of a microfluidic system	Selection can be automated, association with Mag-SELEX or CE-SELEX possible	[5, 7]
HTS-SELEX	Addition of high-throughput sequencing to each round	Early identification of sequence enrichment, leading to a gain in efficiency and a decrease in the number of cycles required	[5, 7, 10]
Cell-SELEX	Cells used as targets, with a negative selection step using healthy cells	The target is a transmembrane protein or an unidentified protein specific to a cell type	[5, 7, 8, 11]
In vivo SELEX	Selection in living organisms by injection of nuclease-resistant nucleic acids	Selection of aptamers that target a tissue and can penetrate or identify it in a living organism, identification of protein markers in a tissue	[5, 7]
Spiegelmer	For chiral targets, SELEX process performed on the enantiomeric form of the target, then synthesis of the selected aptamer with l-nucleotides	More stable and nuclease-resistant aptamers	[5, 8]
Chimeric SELEX	Selection of two aptamers specific of two targets, and combination in a single molecule	Aptamer that can bind to two separate targets	[5]
Toggle-SELEX	Use of homologous targets, from different species, every other round	Aptamer with significant affinity for its target even when changing species	[5]
Crossover-SELEX	Use of purified protein or tissue/cell as targets every other round	The selected aptamer has more chance to recognize its target in vivo	[5]
Truncation SELEX	All probable nucleotide truncations of the selected aptamer are used to perform a new screening	The size of the final aptamer is reduced while being as much or more efficient than the initial one	[5]
Modified SELEX	Use of modified nucleotides	Modification of aptamer properties	[5]

### Unmodified aptamer properties

It is important to first present the properties of unmodified aptamers to understand why modifications are applied in certain fields of application. The length of aptamers generally varies between 20 and 100 nucleotides. However, short aptamers of 8 nucleotides or long aptamers of up to 228 nucleotides have been developed [12]. Aptamers are generally small molecules that can range from 6 to 40 kDa and from 1 to 2 nm in diameter [13]. As nucleic acids, they are susceptible to degradation by nucleases. Natural nucleic acids survive for 5 min in serum and 1 h in living cells [12]. Aptamers are no different and, in vivo, they are affected by endonucleases and exonucleases. For instance, a DNA aptamer selected in 1993 targeting thrombin has an estimated in vivo half-life of 108 s [14]; similarly, an unmodified RNA aptamer binding human keratinocyte growth factor (hKGF) has a half-life of less than 8 s in 90% human serum [15].

Aptamers are considered thermostable. A significant increase in temperature can however disrupt the secondary and tertiary structure of the aptamer and accelerate its degradation by nucleases, or even lead to its complete denaturation [12]. Nonetheless, aptamers have the ability to return spontaneously to their initial conformation after being denatured by heat: their thermal denaturation is reversible [16]. For example, the theophylline RNA aptamer mTCT8-4 has a melting temperature T_m_ of 72 °C, at which 50% of the RNAs are denatured [17].

Owing to the usual equipment available in laboratories, aptamers can be easily handled, stored, but also immobilized and labeled [13]. New chemical functionalities can be easily added through conjugation and addition of moieties at specific sites [18].

As short-sequence nucleic acids, aptamers possess complex pharmacokinetic characteristics that are generally not suited for the development of therapeutic tools. Aptamers made only of unmodified nucleic acids are distributed rapidly and widely from the plasma compartment to the tissues, with the highest concentrations in the liver, kidneys, lymph nodes, spleen and bone marrow [19]. Their half-life in blood is very short, around 1 min, particularly due to the action of nucleases in plasma [18]. Aptamers are small enough to easily penetrate biological barriers, giving them access to most compartments. However, because their size rarely exceeds 30 kDa, aptamers are also filtered by the renal glomerulus and are therefore rapidly eliminated from the bloodstream. Despite a wide and rapid distribution to tissues, the sensitivity to nucleases and rapid renal filtration lead to a very limited bioavailability of unmodified aptamers. This is a significant limitation to their use in vivo, particularly as therapeutic molecules. Overall, aptamers are considered to be safe. Toxicological studies with aptamers have not reported any direct activation of the immune system or the complement system [19].

Aptamers are very often compared to monoclonal antibodies in different fields of application. Overall, they are tools that exhibit high affinity and specificity towards a defined target. To understand better the advantages and disadvantages of these two tools, their properties as well as their manufacturing and storage characteristics are collated in Table 2 [1, 13, 18, 20–23]. Table 2 shows the advantages of aptamers over monoclonal antibodies when it comes to production, manipulation, modifications and storage, although antibodies possess more suitable pharmacokinetic properties for in vivo applications.

**Table 2:** **Properties of aptamers versus monoclonal antibodies**

Criteria	Aptamers	Monoclonal antibodies
Molecular weight/size	Low molecular weight: 6–30 kDa (20–100 nucleotides) Around 2 nm	High molecular weight: 150–180 kDa Around 15 nm
Potential targets	Wide range of possible targets: ions, organic molecules, nucleic acids, amino acids, carbohydrates, antibiotics, peptides, toxins, cells, …	Only immunogenic molecules Few toxins
Generation and manufacture	In vitro SELEX Around 2 to 8 weeks, but can be hours via HTS-SELEX Cost of selection around 4000$ Low risk of contamination Possible large-scale production The environmental parameters of selection can be modified	In vivo biological system Around 6 months or longer Cost of selection around 8000$ for mouse antibody, and 20 000$ for rabbit antibody Potential contamination due to cells or animal-based production Large-scale production not available without deteriorating the quality of the final product The environmental parameters must match the physiological environment
Reproductibility	High reproducibility, no batch-to-batch variation	Significant batch-to-batch variation
Conservation	Long shelf-life and stability Can be lyophilized, easily transported and stored at room temperature	Limited shelf life, unstable Must be cooled for transportation and storage
Physical and thermal stability	Resistant to high temperature Can be reversibly denatured Reusable	Susceptible to temperature (even at Room temperature or 37 °C) Susceptible to irreversible denaturation Single use
Chemical modification	Wide variety of chemical modifications that are site-specific and easily performed during synthetis or before selection	Chemical modifications are limited, not site-specific and inconstant
Immunogenicity	None or low	High
Pharmacokinetics (tissue uptake, kidney filtration, nuclease degradation)	Efficient entry into biological compartments, susceptible to renal filtration and nucleases Short circulating half-life Pharmacokinetic properties can be improved	Limited access to many biological compartments, not susceptible to nucleases nor renal filtration Long circulating half-life Pharmacokinetic are not easily modified
Specific antidote	Yes	No

References: [1, 13, 18, 20–23]

### Aptamer modifications

**Figure 2:**
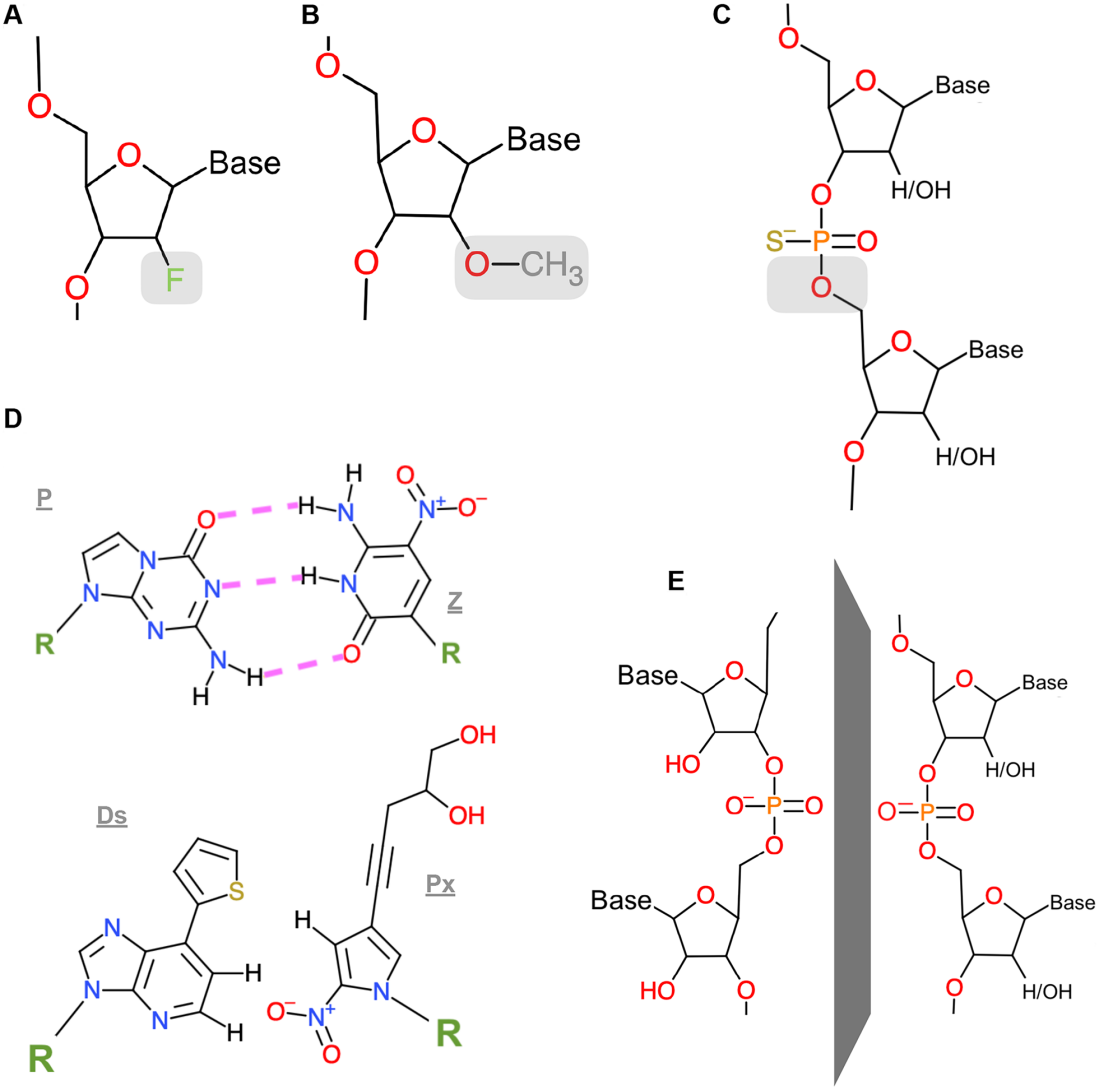
**Examples of aptamer modifications.** Chemical structures of modified aptamers. **A** XNA 2′-fluoro-ARN, **B** 2′-methoxy-ARN, **C** α-phosphorothioate, **D** unnatural bases P:Z and Ds:Px, **E** Spiegelmer as the mirror image of an aptamer made up of wild-type nucleic acids.

The limits of unmodified aptamers mentioned above, in particular the properties which they lack in comparison to antibodies, can be overcome with chemical modifications. These modifications are numerous and varied. They can be classified according to whether they are carried out before or after SELEX, but also according to their location on the nucleotides or on the sequence. The consequences of such modifications on aptamer properties are summarized in Table 3 and the modifications given below as examples are shown in Figure 2.

**Table 3:** **Consequences of chemical modifications of aptamers**

Location of the chemical modification	Examples	Consequences
Backbone	α-Phosphorothioate	Nuclease-resistance
Sugar	XNA 2′-Fluoro-RNA 2′-Methoxy-RNA	Nuclease-resistance
Nucleobase	C5 Unnatural base	Increased chemical diversity Higher affinity
End of sequence	PEGylation	Reduced renal filtration

The backbone of a nucleotide triphosphate can be modified at any of the three phosphorous atoms or at the oxygen atoms. The modifications used are not very diverse and the most common one leads to an α-phosphorothioate [12]. This modification consists in replacing an oxygen atom with a sulfur atom on the α-phosphorus of the nucleotide triphosphate. Among other things, it has the advantage of being compatible with the enzymes involved in the SELEX process and is therefore used in the design of libraries. It confers nuclease resistance to the resulting aptamers that can still be taken up by cells [24].

Modifications can also be applied to the sugar unit of nucleotides and offer many possibilities. The resulting nucleic acids have been coined XNA for “xeno-nucleic acids”. XNAs are defined as synthetic genetic polymers whose sugar is neither a deoxyribose nor a ribose [25]. XNAs are generally generated post-SELEX. However, some modification techniques can occur before selection, resulting in a selection process called X-SELEX, which uses mutant polymerases that accept XNAs [26].

Sugar modifications are mainly used to make nuclease-resistant aptamers. For this result, they most often intervene in position 2′ of the ribose, because most ribonucleases polarize the 2′ hydroxyl group to attack the phosphodiester bond and hydrolyze RNA sequences [27]. The most frequently substituted 2′ groups are amine (NH_2_), fluorine (F) and methoxyl (OCH_3_). Aptamers made up of nucleotides with modified 2′ groups continue to have high affinity for their target and exhibit high resistance to nucleases [24].

Along with the sugar unit, the nitrogen bases can undergo the most important modifications. Pyrimidine bases are modified in the C5 position, and purine bases undergo modifications in N7. These positions are good substrates for polymerases and do not interfere with complementary base pairing [24]. These modifications can also be carried out before SELEX.

Adding functional groups to the nitrogen base can improve the affinity of an aptamer for its target by increasing the surface area of interaction or by creating new secondary and tertiary configurations [12]. It can also increase the diversity of targets against which aptamers can be selected, such as hydrophobic or negatively charged molecules known to be difficult for the selection of aptamers [28].

Another category of aptamers with modified nucleobases comes from research on the evolution of genetic systems, which led to the creation of artificial bases. Kimoto et al*.* introduced a third exclusive base pair: 7-(2-thienyl)imidazo[4,5-b]-pyridine (Ds) and 2-nitro-4-propynylpyrrole (Px). This Ds:Px pair has yielded aptamers targeting interferon γ and vascular endothelial growth factor 165 (VEGF-165), with affinities 100 times higher than those of aptamers made up of only natural nucleotides [29]. There are other artificial bases such as the Z:P pair (with Z = 6-amino-5-nitro-2(1*H*)-pyridone and P = 2-amino-imidazo[1,2-a]-1,3,5-triazin-4(8*H*)-one) which also induces high affinity [24].

Spiegelmers (obtained by Spiegelmer SELEX) are mirror aptamers, made up of l-nucleotides, i.e., the plane mirror image of naturally occurring nucleotides. They are resistant to nucleases, therefore very stable in plasma, and immunologically passive. They are mainly used in clinical research as potential drugs [30].

Due to the small size of aptamers, modifications of the nucleobases or of the sugar-phosphate backbone generally do not affect renal filtration. The strategy most frequently used to counter this problem is the conjugation of a large molecule to the aptamer to increase its mass to more than 30–50 kDa and thus avoid filtration by the renal glomerulus. The most commonly used modification is the addition of polyethylene glycol (PEG) units weighing 20 or 40 kDa. PEG is an amphiphilic polymer with a half-life of 10 h in the circulating plasma of mice. It is frequently used to increase the bioavailability of a drug and it is non-toxic at the concentrations used. Conjugation occurs at the 3′ or 5′ end of the aptamer and is called “PEGylation”. The best-known example of a PEGylated aptamer is pegaptanib, the only aptamer approved by the Food and Drug Administration (FDA), and will be discussed below [12]. However, some antibodies directed against PEG have been discovered, and their presence can lead to side effects such as hypersensitivity reactions in some patients [19].

Aptamers thus possess properties that are very useful for biological recognition. They can be used similarly to antibodies as biosensors. A biosensor is an analytical tool that combines a bioreceptor and a transducer [23]. The bioreceptor recognizes a target and binds to it with high sensitivity and specificity, while avoiding interference with other molecules or microorganisms. The transducer translates the binding between the analyte and the bioreceptor by sending biological signals. Aptamer-based biosensors are referred to as “aptasensors”. They can include an aptamer as a bioreceptor or more rarely an aptamer as a transducer. The use of aptamers offers many advantages because they can be easily modified, especially for the addition of signaling groups.

**Figure 3:**
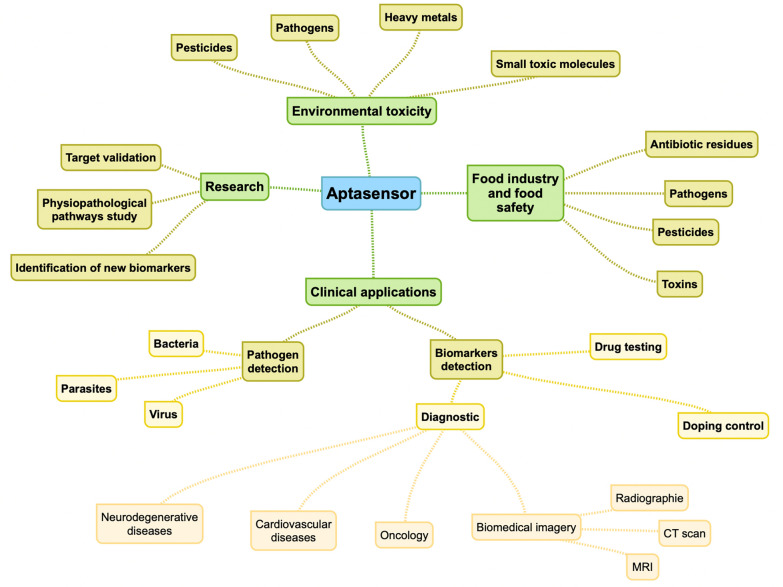
**Applications of aptasensors.** Mind map: fields of applications are shown in green, examples within those fields are shown in yellow.

The applications of aptasensors are as varied as the types of targets for which they are created. They can be used for rapid and accurate detection of disease markers, pathogens, antibiotics, toxins, pollutants or any other chemical compound or biomolecule [31]. For this reason, they are used in basic research for the study of physiological or pathological pathways and infectious diseases, but also in medicine for the detection of pathogens or tumor cells. They are also widely used in the study of environmental contaminants, particularly in water or food. Figure 3 summarizes the fields of application of aptasensors and gives some examples.

## Applications in food and environmental safety

### Detection of pathogens and toxins

Foodborne pathogens are numerous. Most of them are bacteria, but they can also be viruses or parasites. Some of these pathogens also produce toxins that cause food poisoning. The detection of foodborne pathogens relies on various methods: culture, immunoassays, PCR, genetic markers and biosensors [32].

Aptasensors have already been selected against many microorganisms involved in foodborne illnesses. For instance, aptamers have been selected against *Escherichia coli* O157:H7 [33], *Salmonella enterica* serovar Typhimurium [34], *Staphylococcus aureus* [35], *Campylobacter* spp. [36], *Listeria monocytogenes* [37], *Shigella dysenteriae* [38], *Vibrio vulnificus* [39] and *Vibrio parahaemolyticus* [40].

In 2018, Zou et al. selected a DNA aptamer named “Apt-5” after 14 rounds of cell-SELEX with different stages of *E. coli* O157:H7 [41]. Enterohemorrhagic *E. coli* (EHEC) can cause severe hemolytic uremic syndrome. Under the assumption that the structure of the bacterial outer membrane may vary between growth phases, *E. coli* at different phases were used as targets: adjustment phase, log phase and stationary phase. Three cycles of counter-SELEX were also added, involving other pathogens, including an enterotoxigenic *E. coli* (ETEC), thus enhancing the specificity of the selected aptamers. Among five DNA sequences selected at the end of the process, the Apt-5 sequence was determined to be optimal with the lowest K_d_ value and the best specificity. Finally, other experiments were performed to study the binding mechanism of the aptamer to the bacteria, suggesting that Apt-5 targets lipopolysaccharides (LPS) [41].

Other important pathogens involved in foodborne illnesses are noroviruses of the Caliciviridae family, which cause acute gastroenteritis in humans [42]. Their culture is impossible, making their detection difficult. The only detection method currently in use is quantitative RT-PCR. There is therefore a real need for norovirus detection tools that do not require specialized equipment. In 2018, Schilling et al. selected a new aptamer against noroviruses that could be used to extract and detect viral particles in food matrices. Several SELEX methods with different matrices were used to select aptamers: buffer solution alone, a suspension of lettuce, a suspension of strawberries and a suspension of oysters. The presence of food in the matrix did not facilitate the selection of DNA sequences with good affinity and specificity. In particular, the strawberry SELEX experiment resulted in the absence of DNA particles after nine cycles, and selection in the oyster suspension did not lead to the enrichment of oligonucleotides at all. Thus, the best aptamer candidate for noroviruses was found to be “Buf-2”, a DNA sequence selected in buffer solution alone. This aptamer targets the P-domain (for “protruding domain”), a part of the major capsid protein. The study of aptamer P-domain binding in different matrices shows a constant binding intensity despite the addition of food and although this aptamer had been selected without a food matrix [42].

Few aptamers have been selected against parasites that can be found in food, most of them being protozoa. In 2015, Iqbal et al. selected a DNA aptamer targeting the wall of *Cryptosporidium parvum* oocysts [43]. They designed an electrochemical aptasensor that can quantify the number of oocysts in the range of 150 and 800 oocysts, with a detection limit of approximately 100 oocysts. The sensitivity and specificity of the aptasensor was then successfully tested on pineapple and mango concentrates [43].

Finally, one of the advantages of aptamers over antibodies is their ability to be selected against any toxin, which can be useful in food safety. Among the common foodborne illnesses, some are due to poisoning, as it is the case with *S. aureus* enterotoxins, which are heat stable and toxic from 100 ng [44]. In 2012, DeGrasse selected a DNA aptamer specifically targeting staphylococcal enterotoxin B, called APTSEB1, which does not bind to other staphylococcal enterotoxins [44]. However, no dissociation constant was reported. Aptamers have also been selected against other biotoxins such as ricin, abrin or cholera toxin [45]. Kirby et al. used an aptamer against ricin to create a detection system based on streptavidin agarose beads and demonstrated its ability to be reused [46]. They used fluorophore-labeled ricin to detect binding to the aptamer beads. The aptamers were denatured thermally 3 min at 70 °C, or by washing with a solution containing 7 mol/L of urea, or both. After denaturation, the system showed no loss in efficiency, thus demonstrating the possibility for reuse [46].

### Detection of drugs

The presence of antibiotic residues in food is an important factor in the issue of antibiotic resistance. Aptamers hold promise for use in detecting antibiotics or their metabolites in food, thereby reducing the necessary time, equipment and labor [47]. Aptasensors have thus been developed to detect the presence of β-lactams (penicillin, ampicillin), aminoglycosides (streptomycin, gentamicin, kanamycin, neomycin, tobramycin), anthracyclines (daunomycin), chloramphenicol, fluoroquinolones (ciprofloxacin, danofloxacin, enrofloxacin, ofloxacin), lincosamides (lincomycin), tetracyclines (tetracycline, oxytetracycline) and sulfonamides (sulfadimethoxine) [48].

In 2018, Sadeghi et al*.* selected a DNA aptamer directed against florfenicol and its dominant metabolite, florfenicol amine, to detect them in raw milk [49]. Twelve cycles of SELEX resulted in several candidates, including the aptamer Apt125. With this aptamer, they created a fluorescent aptasensor that can detect up to 5.75 nmol/L of florfenicol in 90 min [49]. Another foodstuff that can frequently contain antibiotic residues is honey. The situation is less common in the European Union, an area where treating bees with antibiotics is prohibited. In America, it is not unusual to treat bees with oxytetracycline to combat European foulbrood. Wang et al. thus designed a competitive direct enzyme-linked aptamer assay (ELAA) test to detect up to 0.098 ng/mL of oxytetracycline in honey [50].

In addition to pathogens and antibiotics, other potentially harmful substances for consumers can be found in food, such as endocrine disruptors. These are most often artificial molecules that can have long-term harmful effects by interfering with the endocrine system [51]. For instance, the European Union has banned bisphenol A use in baby bottles since 2011. Nonetheless, bisphenol A is still used in the composition of certain plastics. In 2014, Zhou et al. developed an electrochemical aptasensor to detect bisphenol A present in liquid milk and powdered milk samples with a detection limit of 5 nM [52].

It is possible to detect other substances sometimes used fraudulently such as melamine—a resin component with renal toxicity, used in the past to artificially increase the measurement of the protein content in foodstuffs—or hormones administered to animals to increase productivity [45].

### Detection of pollutants

Contamination by industrial pollutants and pesticides is a real problem due to their increasing quantities. Among the industrial pollutants for which there is an aptamer, it is possible to cite bisphenol A and melamine already mentioned above, as well as polychlorinated biphenyls (PCBs), synthetic compounds formerly used as lubricants and coolants in industry. The production of PCBs has been banned for over 10 years because they are extremely toxic and carcinogenic [53]. However, due to their long persistence in the environment, the need for a detection method is still present. Given that PCBs constitute a group of 209 molecules, aptamers have been selected to recognize several members of this class with a single aptasensor [54]. Selection was based on two targets, PCB 72 and PCB 106, with a dissociation constant between 60 and 100 nM. In addition to these targets, the selected aptamer also exhibits significant affinity for other PCBs, notably PCB 105, PCB 118, PCB 153 and PCB 169, although to a lesser extent than for its initial targets.

Another consequence of industrialization is environmental pollution from heavy metals. Metal ions are not biodegradable and exhibit a wide variety of toxicities [47]. In 2019, Mao et al. carried out an inventory of nanomaterial-based aptasensors capable of detecting the presence of arsenic, in particular by means of portable tools [55]. Aptamers targeting lead have also been selected, including a thionine and graphene-based aptasensor with an estimated detection limit of 3.2 × 10^–14^ M [56]. The most studied metal element is mercury. In 2017, Zeng et al. designed a reusable electrochemical sensor capable of detecting up to 3.6 × 10^–12^ M of mercury ion in drinking water, river water or runoff water. This sensor can be used for on-site detection [57].

Finally, aptamers have been selected to detect pesticides, such as organophosphates, important agricultural pollutants. In 2012, Wang et al*.* selected two DNA aptamers directed against four organophosphates (phorate, profenofos, benzoate and omethoate) [58]. These aptamers can be used for pesticide detection, and can also neutralize these pesticides [58].

## Diagnostic applications in veterinary medicine

Aptamers can potentially be used to create point-of-care tests in individual and livestock pathology. The creation of cheap tests is also an interesting prospect to facilitate access to medical examinations. We focus here mainly on infectious diseases, because the existing tools in human medicine can be the most easily transposed to veterinary purposes. Moreover, based on the number of articles published, this field is growing rapidly and offers the most applications for aptamers. Other applications such as cardiovascular diseases or cancer will be mentioned more briefly, because they do not show as much potential in veterinary medicine as in human medicine.

### Infectious diseases

Aptamers can be used to detect pathogens in biological samples. The list of these targets is long and includes viruses, bacteria, parasites, and also markers of inflammation or antibodies that indirectly demonstrate the presence of an infection. Many infectious diseases are zoonoses, making the existing applications in human medicine easily transposable to veterinary medicine. In addition, some pathogens found in human medicine belong to the same family as other pathogens common in veterinary medicine.

#### Viral infections

The diagnosis of viral infections is the most widely studied application of aptamers in veterinary medicine. In the vast majority of cases, these viruses affect livestock and are particularly contagious, with high morbidity and sometimes mortality rates, leading to dramatic economic losses for the sector. Several influenza viruses of the viral family Orthomyxoviridae are the targets of aptamers, including H1N1, H3N2, H5N1 or H9N2 [23]. Some of these viruses are ubiquitous and dreaded in poultry or pig farms, such as avian flu or swine flu. Past health crises justify research on efficient and rapid diagnostic tools that can detect the circulation of influenza viruses on farms and identify their subtype, particularly considering the significant contagiousness of some of them. For example, the highly pathogenic avian influenza virus H5N1 was studied by Pang et al. in 2015 following the devastating epidemics that led to high mortality rates in poultry populations, but also in humans [59]. The diagnosis is usually established by PCR or immunological tests, requiring the transport of samples and their analysis in laboratories equipped with suitable machines used by specialized personnel. It is therefore of interest to develop detection methods that can be used by professionals in the field, and that give immediate results, in particular during an epidemic. Rapidity is all the more important for influenza viruses that cause very unspecific symptoms. Pang et al. designed a fluorescent aptasensor selected against a recombinant hemagglutinin of the H5N1 virus [59]. This biosensor has a limit of detection (LOD) of 2 ng/mL in buffer, and 3.5 ng/mL in a complex matrix with human serum. The use of a portable spectrophotometer may allow detection of the H5N1 virus on site [59]. Similarly, two years later, Wongphatcharachai et al. selected four DNA aptamers targeting hemagglutinin subtype H3N2 circulating in pigs in North America [60]. Among these four aptamers, one has the particularity of binding to several hemagglutinins H3 from different lineages, including lineages that can affect birds or humans [60].

Another devastating virus is the foot-and-mouth disease (FMD) virus, a Picornaviridae which caused the 2001 and 2007 epizootics in Great Britain. It can affect cattle, sheep, goats and pigs, with varied and relatively non-specific clinical signs that can be confused with other less serious conditions. The suspicion of an outbreak of FMD can have costly and drastic consequences for a sector and a country. The existence of a rapid and portable test that can be performed in the field would be a real advantage, saving considerable time and money given the threat posed by this virus. In 2008, Bruno et al. sought to develop an aptamer-based diagnostic test, allowing the detection of a peptide from the VP1 structural protein [61]. The use of a polyclonal family of aptamers provided a signal-off response in about 10 min, with a sensitivity of 250 ng/mL for the VP1 peptide. The selection of this polyclonal family allowed the cloning and sequencing of each aptamer, with some exhibiting high affinity towards the FMD peptide. However, it has not been possible to match the sensitivity of the polyclonal family using a single aptamer. Indeed, affinity is not the only factor that can influence the sensitivity of a test. In this case, the test is based on a competitive assay in which the aptamers are labeled with a fluorophore: the position and the number of fluorophores incorporated being of great influence [61].

Bluetongue virus (BTV) is an orbivirus of the Reoviridae family at the center of recent health crises, with severe economic repercussions. The several serotypes, which can co-exist in the same area, and the use of vaccination in certain areas complicate the diagnosis. There is currently no aptamer directed against a serotype of BTV, but in 2009, Danielli et al. designed a tool for the detection of Ibaraki virus, a strain of an epizootic hemorrhagic disease virus belonging to the same viral family as BTV and producing similar clinical signs [62]. This device detected 1.9 pM of viral DNA in just 18 min [62]. Based on that example, a similar tool could be developed to detect the different serotypes of BTV.

Other aptamers have also been selected against a wide variety of viruses, many of them being zoonotic: notably aptamers against rabies virus of the Rhabdoviridae family [63], vaccinia virus of the Poxviridae family [9], human immunodeficiency virus of the Retroviridae family [64], noroviruses of the Caliciviridae family [42], the SARS-CoV coronavirus of the Coronaviridae family [65], the bovine respiratory syncytial virus of the Paramyxoviridae family [66] and the Muscovy duck parvovirus [67].

Based on these studies in livestock, similar tools could be developed for pets. For example, canine distemper is caused by a virus from the same family as the bovine respiratory syncytial virus. Likewise, some common parvoviruses can be fatal to young dogs and cats. Finally, feline infectious peritonitis (FIP) is another disease for which there is a real need for diagnostic tools. It is caused by a mutated enteric coronavirus. Diagnosis is particularly difficult in dry forms, and there is still no ideal diagnostic test: serology does not distinguish between antibodies developed against enteric coronavirus from antibodies to FIP; the specificity of RT-PCR in any given sample is not absolute; S gene mutations are not constant and are sometimes found in enteric coronaviruses. Histology and immunohistochemistry remain the most reliable tests, but they are most often performed *postmortem*. Using aptamers may be the solution to distinguish the FIP virus from the feline enteric coronavirus thus enhancing the specificity of the available tests, or they could be used to create new biosensors that would increase the sensitivity of the tests performed on aqueous humor or cerebrospinal fluid [68].

#### Bacterial infections and intoxications

The diagnostic tools for bacterial infections in humans are generally transposable to animals. Bacteria involved in food poisoning can be tested for in livestock. They are not always pathogenic, but carriage by animals whose flesh is intended for human consumption can pose a health risk. Thus, in 2011, Gnanaprakasa et al. designed a DNA biosensor to detect the *hipO* gene of *Campylobacter jejuni* [69]. They combined it with diffraction optics technology (DOT) or surface plasmon resonance (SPR) transduction platforms, offering respectively a LOD of 5 nM and 2.5 nM. The SPR sensor could even be re-used several times without significant variation in the efficiency of the test [69].

In 2014, Fang et al. designed a lateral flow biosensor to detect *Salmonella enterica* serovar Enteritidis [70]. The test was made up of two aptamers: a capture aptamer (c-aptamer) and an amplifying aptamer (a-aptamer). It detects as little as 10^1^ colony forming units (CFU) of bacteria very specifically, because it does not detect other serovars of *S. enterica*, or other bacteria [70]. Not all *S. enterica* serovars are classified as health hazards, explaining the need to distinguish them via a rapid test.

Finally, an indirect competitive ELAA test allows the detection of antibodies against *Mycoplasma bovis* in bovine serum [71]. This test uses the WKB-14 aptamer which targets the *M. bovis* P48 protein. In the presence of antibodies in serum, competition for the P48 protein results in a decrease in the optical signal. The results show sensitivity and specificity similar to the direct competitive enzyme-linked immunosorbent assay (ELISA), and better sensitivity than two marketed indirect ELISA kits. The specificity was demonstrated through cross-reactivity with antisera of other related pathogens that all gave negative results [71].

Some veterinary diseases are caused by the entry of toxins into the body. *Antemortem* diagnosis is most often clinical and based on the patient’s history, and *postmortem* diagnosis is usually based on the exclusion of other diseases in the absence of any significant lesions. Potent neurotoxins that can contaminate food are botulinum toxins produced by *Clostridium botulinum*. These toxins lead to flaccid paralysis and death. The detection of these toxins is difficult, which is why it is complicated to establish a definite diagnosis. The method routinely used is the in vivo mouse lethality bioassay and cross-neutralization test using antibodies specific to serotypes [72]. In 2012, Bruno et al. selected a DNA aptamer associated with a fluorescent signal to detect botulinum toxins [73]. They selected it using botulinum toxin A as a target and, after 10 rounds of SELEX, obtained an aptamer called “Beacon 10” with a LOD of 1 ng/mL. Bruno et al*.* also explored the possible use of this aptamer for on-site detection of toxins in diluted soil suspensions with a handheld fluorometer. Finally, cross-reaction tests have shown a weaker, but significant, affinity of Beacon 10 for botulinum toxins B and E, which may represent an advantage for rapid environmental detection, but is poorly suited for a medical diagnosis [73].

The detection of botulinum toxins may also be useful in the diagnosis of grass sickness or equine dysautonomia. The main suspected causative agent for this condition is botulinum toxin C [74]. Toxins produced by other *Clostridia* are also frequently involved in diseases, such as *Clostridium perfringens* enterotoxaemia in sheep, which progresses rapidly and is often fatal. Nonetheless, detection of the involved toxin and its typing is hardly ever done in the field; diagnosis remains also complicated due to the presence of *Clostridia* in healthy animals [75].

#### Parasitic infections

Cryptosporidiosis found in humans is a disease causing neonatal diarrhea in calves, lambs and kids. The most frequent species is *C. parvum*, for which Iqbal et al. designed an aptasensor initially intended to identify oocysts on food [43]. If the sensor proves able to identify oocysts in a complex environment such as feces, the on-site diagnosis of cryptosporidiosis may be facilitated on farms. Other subclass Coccidia parasites frequently encountered in veterinary medicine are *Eimeria* spp. and *Isospora* spp. respectively affecting herbivores and carnivores. An aptamer could be developed to detect oocysts of these coccidia in feces.

Likewise, schistosomiasis is a zoonotic parasitic disease for which many wild and domestic animal species can act as a reservoir. *Schistosoma japonicum* can be found in cattle, pigs, sheep and dogs. Infestation of farm animals by *S. japonicum* can also lead to economic losses [76]. In 2016, Long et al*.* identified two aptamers LC6 and LC15 capable of recognizing *S. japonicum* eggs specifically, without affinity for the eggs of other parasites such as *Fasciolopsis buski*, *Enterobius*, *Ascaris* or *Clonorchis sinensis* [77]. The LC15 aptamer may even reveal eggs within liver tissue samples with a detection rate of 80.5%. Aptamers LC6 and LC15 are potential tools for detecting egg-shedding animals [77].

Finally, useful applications of aptamers include those directed against *Giardia duodenalis*, which causes diarrhea in domestic carnivores, as well as against *Toxoplasma gondii*. The latter is the causative agent of toxoplasmosis, a ubiquitous disease of importance in human medicine due to its severity for the fetus in pregnant women and for immunocompromised people. Its definitive host is a Felidae, most often a cat. Diagnosis in cats is complicated, especially when wishing to determine whether the cat is infected or to assess oocyst excretion for risk evaluation. Aptamers may, for example, allow the development of more sensitive tools to assess the excretion of oocysts [78].

#### Poisoning

The diagnosis of poisoning in veterinary medicine is most often based on medical history and clinical evaluation. Poisoning is relatively frequent. The toxic agents most often involved vary with the animal species. Regarding livestock, the main cause is heavy metal poisoning, especially lead for cattle, and copper for sheep and goats [79]. Regarding dogs, the toxic agents are mainly rodenticides (such as strychnine or anticoagulants) and acetylcholinesterase inhibitors such as organophosphate or carbamate insecticides.

The search for a toxic agent can take place in an evocative context or as part of a necropsy with no lesions of clinical significance. Several aptamers have already been tested successfully among the main toxins to look for. This is for example the case with lead [56] or organophosphorus compounds [58]. The problem remains to be able to show the specificity of these aptamers for their target in a complex medium such as biological samples.

#### Prionopathies

Aptamers have been developed to diagnose prionopathies (also named transmissible spongiform encephalopathies or prion diseases), in particular Creutzfeldt-Jakob disease and kuru disease in humans, scrapie in sheep and bovine spongiform encephalopathy in cattle. Aptamers have been selected against several prions of different conformations and different species, including hamster, cow, sheep and human [80]. In 2010, Xiao et al. designed an aptamer-based tool to detect the presence of the prion protein PrP^Res^ (resistant to proteases) in serum and brain samples, and above all to differentiate it from the PrP^C^ protein (normal cellular prion protein) [81].

### Oncology

Selecting aptamers using cell-SELEX can provide biosensor tools specific to a cell type, including tumor cells. These tumor cells carry biomarkers on their surface that distinguish them from other cells. Tumor diagnosis is most often based on histomorphological analysis, sometimes combined with other technologies such as immunohistochemistry or molecular profiling [82]. For lymphomas, the diagnosis particularly includes immunophenotyping, karyotyping and detection of mutations by PCR [83]. Aptamers have already been selected against a wide variety of tumor cells and biomarkers.

The use of aptamers in combination with medical imaging techniques is also possible. The selection of cell type-specific aptamers via cell-SELEX may allow the identification of any tumor tissue, even in ambiguous cases where histological examination is not conclusive. As in human medicine, aptamers may replace lymphoma cell immunophenotyping techniques in the diagnosis of lymphoma and leukemia. With feline low-grade alimentary lymphoma, histological analysis of intestinal biopsy samples is often combined with immunohistochemistry or even clonality testing, although the results are not always clear-cut, making it difficult to distinguish from inflammatory bowel disease [84]. The use of anti-CD3 aptamers may, for example, facilitate diagnosis and replace anti-CD3 antibodies. Finally, future research on aptamers may identify new markers specific to low-grade alimentary lymphoma.

### Cardiovascular diseases

The aptamers studied in human medicine revolve around the development of atheromatous plaques or the occurrence of ischemic attacks in the myocardium, which are very infrequent diseases in pets, and are therefore of little use in veterinary practice. Potentially useful aptamers include those that could be selected against the blood biomarkers commonly used in veterinary medicine in the exploration of heart disease, such as troponin I and different forms of the ventricular natriuretic peptide like C-BNP or NT-proBNP [85].

### Other prospects in diagnosis

Aptamers are currently being studied in cattle for the early diagnosis of pregnancy. Very recently, Lu et al. selected DNA aptamers targeting bovine glycoproteins associated with gestation (bPAG) expressed by day 25 [86]. They combined two of these aptamers with SPR techniques and applied them successfully to bovine serum samples. Early pregnancy diagnosis in dairy cattle farms is indeed a key element in the reproduction monitoring carried out by the veterinarian, with large economic consequences for the breeder. It is currently based on transrectal palpation and transrectal ultrasound, with a diagnosis possible as early as 29 days after breeding. However, these are time-consuming methods that require some dexterity and experience on the part of the operator. Although antibody-based immunoassays for detecting bPAGs in blood or milk have already been developed, aptamers could replace them to produce rapid tests that are cheaper, reusable, easier to store and applicable on site. The early detection of a significant increase in bPAGs is required to identify non-pregnant cows, and therefore makes it possible to re-inseminate them as soon as the following estrus. The test developed by Lu et al. produces a colorimetric response with a LOD of 0.037 ng/mL for instrument detection, and 0.134 ng/mL for visual detection. Its sensitivity is sufficient to detect the increase in bPAG that occurs as early as 25 days after breeding [86].

Finally, there are other diagnostic areas that could benefit from the use of aptamers, but for which there are no studies yet. For example, the diagnosis of pancreatitis is sometimes complex, and often requires the combination of medical imaging and the assay of specific pancreatic enzymes. The amount of these pancreatic enzymes can be measured through rapid semi-quantitative assays, that lack sensitivity, or the sample can be sent away to a diagnostic laboratory. Aptamers could be used to select other more specific biomarkers of pancreatitis or to develop more sensitive, quantitative, “pet-side” tests.

## Therapeutic applications in veterinary medicine

Many aptamers, upon binding to their target protein, cause inhibition of its function. For instance, they can prevent their target from interacting with other proteins or from binding to a receptor [18]. In addition to these antagonistic aptamers, there are other modes of action such as agonistic aptamers or aptamer-based targeting ligands. Aptamers directed against proteins involved in pathological pathways may therefore have therapeutic potential [87]. Significant examples are aptamers directed against thrombin, subject of numerous articles that led to some of the first therapeutic applications. Other frequent targets are extracellular protein factors, in particular growth factors such as KGF or VEGF. Therapeutic aptamers are often compared with monoclonal antibodies. They combine the characteristics of antibodies with those of small molecules, such as flexibility and the ability to penetrate tissues. Currently, only one therapeutic aptamer is on the human drug market and more than 30 have reached various stages of clinical trials. Therapeutic aptamers still have a long way to go before they can be used in veterinary medicine. The objective here will therefore be to review the most useful applications that aptamers could offer, after presenting the aptamers on the human drug market.

### Therapeutic aptamers in clinical trials

The majority of the therapeutic aptamers to reach the clinical trial stage target ocular diseases. One reason is that intravitreous injection limits the exposure of the rest of the body to the molecule. In addition, the half-life of aptamers is longer in the eye compared with systemic administration [87].

The first and only aptamer to date to have received marketing authorization (MA) is pegaptanib, registered under the name Macugen®, approved by the FDA in 2004 and by the European Medicines Agency (EMA) in 2006. For comparison, the oldest monoclonal antibody with MA is orthoclone OKT3 and was approved in 1986 by the FDA and in 1988 by the EMA [87]. Pegaptanib is an RNA aptamer targeting and inhibiting VEGF-165, an isoform of vascular endothelial growth factor involved in ocular neovascularization and increased vascular permeability [88]. It is used in the treatment of age-related macular degeneration, the leading cause of blindness in people over 50 years of age. This disease results, among other things, from choroidal neovascularization causing a central visual deficit. In clinical trials, pegaptanib has been shown to reduce vision loss by 50% in the first year of treatment, and to stabilize vision in the second year. Its intravitreous administration is safe, with rare transient and minor side effects more attributable to the injection procedure than to the molecule. However, the high specificity of pegaptanib for VEGF-165 has led to a reduction in its use following the subsequent marketing of other more effective molecules, such as the antibodies bevacizumab and ranibizumab, which inhibit all isoforms of VEGF [87]. Further clinical studies are underway for the application of pegaptanib in some retinopathies, including diabetic macular edema and proliferative diabetic retinopathy [88]. Other aptamers currently in clinical trials include pegpleranib in the treatment of age-related macular degeneration, the anticoagulant pegnivacogin and AS1411 with antiproliferative activity [89].

### Infectious diseases

#### Viral infections

Viral diseases pose a threat to public health, most often due to a lack or limited effectiveness of antiviral treatments. The main protection remains vaccination. However, this does not replace an effective and safe antiviral molecule when needed. Aptamers represent a potential drug that can inhibit certain stages of the viral cycle. Some aptamers prevent the attachment of the viral particle to the host cell, some inhibit viral replication, or more simply some can transport antiviral molecules directly to infected cells [23]. Among the aptamers that can detect viruses, many of them also have shown antiviral activity during experiments. For instance, aptamer C7-35M targeting hemagglutinin H9 from avian influenza H9N2 virus [90] prevents infection of cells during co-incubation of the virus with the aptamer, with increased cell viability compared with the control group of cells incubated with the viral particles alone. Inhibition of viral infection is estimated to be 13% when treated with 100 pmol of C7-35M, and 55% with treatment at 1000 pmol of C7-35 M [90]. In 2015, Zhang et al. performed similar experiments and showed that their aptamers A9 and B4 also inhibited viral infection of cells with H9N2 [91].

In 2014, Forrest et al. selected an RNA aptamer capable of inhibiting the replication of the FMD virus in vitro. This aptamer was selected against 3Dpol, a key enzyme in the virus replication complex, and then chemically modified, but still harboring its initial inhibitory capacity [92].

In 2017, Xu et al. selected a DNA aptamer called IBRV-A4 targeting bovine herpesvirus 1. This herpesvirus is the cause of infectious bovine rhinotracheitis, a disease characterized by damage to the upper respiratory tract, but that can also be responsible for abortions in pregnant cows and encephalitis in calves [93]. The aptamer from Xu et al. may provide a potential diagnostic tool, and also exhibits antiviral properties by preventing the virus from entering cells when administered within 30 min of infection [93].

#### Bacterial infections and intoxications

The search for aptamers to fight bacteria may seem of little use regarding the already existing therapeutic arsenal. However, despite the many families of antibiotics available, the emergence of multidrug-resistant bacteria poses a real threat to public health. New antibiotics are therefore needed to fight against these bacterial infections, often of nosocomial origin and possibly fatal in the absence of treatment [22]. For example, aptamers could be used in combination with antibiotics to counter the resistance developed by bacteria. In 2011, Schlesinger et al. selected aptamers that inhibit β-lactamases, enzymes developed by certain bacteria which have become resistant to β-lactams [94]. More specifically, the study focused on the selection of DNA aptamers targeting a metallo-β-lactamase BcII produced by *Bacillus cereus*. The combination of the selected aptamer and a β-lactam stops bacterial growth, but the antibiotic alone or the aptamer alone have no effect [94].

Another strategy is to select aptamers that target components of bacteria to stimulate passive immunity. In 2008, Bruno et al. selected aptamers directed against the LPS and coupled them to the first component of complement C1qrs to mimic complement fixation [95]. This led to the destruction of bacteria by artificially triggering the natural classical complement cascade. Selecting aptamers against other molecules present on the surface of bacteria may broaden the spectrum of activity. This strategy also has the advantage of inducing little resistance, because bacteria can barely escape attack by the immune system under these conditions [95].

Enterotoxemias are very rapidly developing diseases in farm animals. Symptomatic treatment can be considered only for mild cases. Antitoxins are hardly ever used in the field due to their cost. Likewise, both acute and subacute cases of grass sickness in horses are inevitably fatal [74]. Some aptamers directed against toxins have shown inhibitory effects, in particular against staphylococcal toxins, but also toxins found in snake or spider venoms [22]. For instance, in 2015, Wang et al. selected DNA aptamers antagonists of enterotoxin B, capable of neutralizing it in an in vitro model containing peripheral blood mononuclear cells, and in an in vivo murine model [96]. In the presence of aptamer A11, cytokine release from mononuclear cells is reduced by 64 to 99% depending on the cytokine. This aptamer, when PEGylated and injected into mice 2 h after exposure to the toxin, led to a significant increase in the survival rate compared with the control group [96]. It is therefore plausible to imagine an aptamer that can act as an antitoxin against botulinum toxins, thus making this treatment more accessible.

A serious poisoning in veterinary medicine, and for which an antitoxin aptamer would be very useful, is tetanus. It is caused by tetanus toxins which are produced by *Clostridium tetani* [97]. It is a neurological disease that leads to spastic paralysis and finally death by paralysis of the diaphragm and respiratory muscles. It occurs following contamination of a traumatic or surgical wound by these clostridia. The species most sensitive to the action of these toxins are mainly equines, but also sheep, goats and dogs. As a result, tetanus occurs quite frequently in horses that have not been vaccinated. Treatment includes debridement and wound cleansing, antibiotic therapy and administration of antitetanus serum, made of antitetanus immunoglobulins. A new antitoxin treatment could be created from selecting aptamers that target the tetanus toxin and that also inhibit its action.

#### Parasitic infections

The potential of aptamers as veterinary antiparasitic drugs is based on the examples in human medicine, mainly acting against protozoa. They are mostly tropical diseases that remain relatively rare in animals. For example, Ulrich et al*.* selected several RNA aptamers that bind to host cell matrix receptors on *Trypanosoma cruzi* [98]. Trypanosomiases can affect livestock and therefore have economic repercussions in certain countries of Africa, South America and Asia [99]. Aptamers can be selected against species of trypanosomes other than *T. cruzi*, such as *T. vivax*, *T. congolense* or *T. brucei* and similarly prevent interactions of the parasite with host cells.

In 2009, Niles et al. selected heme-binding aptamers to control *Plasmodium falciparum*, that causes malaria in humans [100]. They were able to show the inhibition of parasitic growth in the presence of these heme-binding aptamers [100]. Other hemoparasites (e.g., *Plasmodium* spp.) include *Babesia* spp. and *Theileria* spp., which cause piroplasmosis. These are serious diseases, sometimes fatal, that can affect ruminants, horses or dogs depending on the involved parasite species. First-line treatment used in animals is imidocarb [101]. However, its use in farm animals is complicated due to long-lasting residues, leading to very long withdrawal periods for milk and meat. The discovery of anti-piroplasmic aptamers could facilitate the treatment of farm animals, because aptamers are molecules with low persistence in the body.

Finally, another common parasitic disease in dogs, caused by a protozoan, is leishmaniasis. Its treatment is also complex since there is currently no molecule that can permanently cure affected dogs. For now, it is based primarily on the administration of allopurinol and meglumine antimonate. It is long and expensive, with notorious side effects, and its effectiveness varies depending on the stage of the disease, which takes into account renal failure and proteinuria [102]. The discovery of a leishmanicidal aptamer could offer a more accessible and less expensive treatment, and may even lead to a parasitological cure.

### Oncology

Oncology is taking an increasingly important place in pet health care, in particular owing to the prolonged lifespan of dogs and cats. It is common practice to diagnose a tumor and the therapeutic possibilities have therefore evolved to approximate the options available in human medicine. In this context, aptamers can play a role similar to their potential in human oncology, but also make the management of cancer patients by their owners more accessible and less expensive.

The ability of aptamers to target a molecule of a signaling pathway specific to a tumor cell is a major asset for therapeutic possibilities. The modes of action are numerous: for example, it could inhibit the proliferation of cancer cells or inhibit angiogenesis. An aptamer specific to a tumor cell line is also an interesting tool for targeted therapies by being coupled to other molecules. Its use depends on the biomarkers specific to each tumor type. Such aptamers conjugated to drugs have been studied with several therapeutic approaches: chemotherapy, immunotherapy, radiotherapy and phototherapy [103]. The challenge is to identify the appropriate target.

Another strategy that can be used is cancer immunotherapy. It is based on the use of monoclonal antibodies, adjuvants or vaccines. Its objective is to stimulate the antitumor immune response and to interrupt the regulatory pathways responsible for immune tolerance towards tumor cells [104]. Not only could aptamers help identify new markers that can serve as therapeutic targets, but they could also replace monoclonal antibodies or be coupled with a component of the complement system to activate an immune response.

### Coagulation

Several proteins involved in the coagulation cascade have been used as targets for aptamers. Whether it is to develop blood thinners, to control coagulation during surgical procedures, or to treat hemophilia, the applications are numerous. In particular, human α thrombin has served as a target for the selection of many aptamers such as HD1, HD22, NU172, RE31 and RA36. It is a multifunctional enzyme that plays an important role in coagulation, homeostasis and inflammation [105]. Other clotting factors have served as targets for aptamers.

Aptamers have also been selected as antiplatelet agents. The existence of an antidote is a real asset. Nimjee et al*.* selected an RNA aptamer targeting the Von Willebrand factor, which participates in the adhesion, activation and aggregation of platelets [106]. They demonstrated its ability to inhibit thrombus formation upon vascular damage in vivo. In addition, the use of a complementary oligonucleotide as an antidote helps reduce intraoperative bleeding [106]. Treatments for bleeding disorders have experienced strong development in veterinary medicine, especially with the arrival of new drugs and an easier access to some molecules for veterinarians. A hypercoagulable state is usually treated with oral antiplatelet agents: aspirin and clopidogrel [107]. Even if they are generally very well tolerated, these molecules irreversibly inhibit platelet function. The use of an aptamer, as developed in humans, may allow better control of the anti-aggregating action, with the possibility of an antidote when the antithrombotic effect is too strong. New injectable molecules inhibiting elements of the coagulation cascade have joined the therapeutic arsenal available to the emergency veterinarian. In particular, low-molecular-weight heparin is more accessible, although further studies are still needed. The need for a safe anticoagulant molecule remains high, and represents a potential application for aptamers.

Finally, there are few veterinary fibrinolytic molecules. These are mainly streptokinase and tissue plasminogen activator, both very rarely used in practice because they are not very accessible, with potentially major side effects and little demonstrated benefit [107]. Developing aptamers with better fibrinolytic efficiency could, for example, make it possible to treat cats suffering from aortic thromboembolism, still too often a reason for euthanasia.

### Autoimmune diseases

Because aptamers are very specific to their target, their applications are often restricted to a particular pathological mechanism or signaling pathway. They may therefore be suitable therapeutic agents for targeting a specific immune response or for inhibiting an inappropriate response, as in autoimmune diseases. They are less suitable for generic use, as an anti-inflammatory drug for example. Aptamers can also be selected against specific antibodies, in particular against autoantibodies involved in certain autoimmune diseases. For instance, Doudna et al. selected an RNA aptamer targeting anti-insulin receptor antibodies that cause severe insulin resistance type B [108]. In addition to strong affinity, it prevents the antibody from binding to the insulin receptor, possibly by acting as a decoy and mimicking the insulin receptor epitope [108]. Similarly, another RNA aptamer has been selected against anti-acetylcholine receptor antibodies, involved in myasthenia gravis. It also acts as a decoy and prevents these autoantibodies from binding to acetylcholine receptors. One limitation, however, is the variability of these antibodies between patients, some of which may not carry the subunit targeted by the aptamer [109].

Autoimmune diseases in veterinary medicine are quite frequent and diverse, particularly in the canine species. Among the most common are immune-mediated hemolytic anemia, immune-mediated thrombocytopenia, acquired myasthenia gravis, immune complex deposition glomerulonephritis, or inflammatory bowel disease [110]. Dogs are also more likely to develop dysimmune polyarthritis or encephalitis, rather than due to an infection. Access to immunomodulatory molecules is therefore of paramount importance, although first-line molecules remain glucocorticoids used in immunosuppressive doses. In addition to glucocorticoids, various immunomodulatory drugs are available, such as azathioprine, ciclosporin or mycophenolate. However, some of these molecules can be expensive, and most may lead to significant side effects. For instance, azathioprine can induce myelosuppression, pancreatitis or liver disease. Aptamers can be useful in diseases where the target of the dysfunctional immune system is known, like myasthenia gravis, mentioned above [109].

Finally, it may be possible to develop an aptamer to replace the monoclonal antibody lokivetmab. It is a “caninised” monoclonal antibody that inhibits interleukin-31, a cytokine involved in chronic inflammation and pruritus. It is currently used in the treatment of canine atopic dermatitis and marketed under the name Cytopoint®. It is highly effective with marked antipruritic action. Its administration is well tolerated with few side effects [111]. Its main disadvantage is its very high cost, despite a low frequency administration with a minimum of 4 weeks between each injection. Particularly in a context of chronic disease for which the animal is treated for life, the price can be prohibitive, hence the interest of an aptamer with the same target, but a much lower production cost.

## Conclusions

The applications of aptamers are exceedingly diverse and fit in a “One Health” approach well. Many diseases in humans and animals could be diagnosed more easily with the use of aptamer-based tools. The high specificity, the selection and manufacturing methods, and the possible chemical modifications of aptamers are all assets for creating diagnostic tests that are accessible “bedside”, convenient, inexpensive, and possibly reusable. Aptamers may also potentially increase the sensitivity and specificity of certain laboratory tests, or be used to create new medical imaging probes. The possibilities seem limitless, and companies have already begun to transform the potential of aptamers into marketable products.

The therapeutic possibilities of aptamers in veterinary medicine are numerous due to a greater need in certain areas compared with human medicine. For example, veterinary medicine must take into account certain limiting factors that may restrict treatment options. For livestock, the price of the molecule and its withdrawal period must be carefully considered. These factors may outweigh the benefit of treating the animal. For pets, the important factors are price, affordability to the veterinarian and owner, side effects, and how easy it can be administered. Finally, the use of certain classes of molecules such as antibiotics or chemotherapy agents is regulated, thus limiting their use. Similar to diagnostic applications, therapeutic aptamers suffer from their excessive specificity. They are often seen as molecules intended for niche markets. However, their great versatility, amenability to modification and moderate manufacturing costs are assets that make them likely to join the veterinary therapeutic arsenal, that is still lacking in certain areas compared to human therapeutics.

With human health being a growing field for the application for aptamers, veterinary medicine can also benefit from this progress. Ideas for veterinary applications have been proposed here in analogy with human medicine or according to current needs. In particular, applications on infectious diseases were emphasized because they offer the greatest potential for immediate or short-term use. Indeed, the majority of research on aptamers focuses on areas that are often shared between human and veterinary medicine, and aptamers may be in high demand in coming years. In other areas, this review revealed the lack of studies on aptamers that could be used by veterinarians, despite the immense potential and growing ideas for human health. Finally, existing aptamers are not always correctly characterized, and most articles would benefit from adding specificity experiments and comparisons to current tests.

## Abbreviations

bPAG: Bovine glycoproteins associated with gestation
BTV: Bluetongue virus
CE-SELEX: Capillary electrophoresis SELEX
CFU: Colony forming units
DNA: Deoxyribonucleic acid
DOT: Diffraction optics technology
Ds: 7-(2-Thienyl)imidazo[4,5-b]-pyridine
EDTA: Ethylenediaminetetraacetic acid
EHEC: Enterohemorrhagic *Escherichia coli*
ELAA: Enzyme-linked aptamer assay
ELISA: Enzyme-linked immunosorbent assay
EMA: European Medicines Agency
ETEC: Enterotoxigenic *Escherichia coli*
FDA: Food and Drug Administration
FMD: Foot-and-mouth disease
FIP: Feline infectious peritonitis
K_d_: Dissociation constant
KGF: Keratinocyte growth factor
LOD: Limit of detection
LPS: Lipopolysaccharide
P: 2-Amino-imidazo[1,2-a]-1,3,5-triazin-4(8*H*)-one
PCB: Polychlorinated biphenyl
PCR: Polymerase chain reaction
PEG: Polyethylene glycol
PrP: Prion protein
Px: 2-Nitro-4-propynylpyrrole
RNA: Ribonucleic acid
RT-PCR: Reverse transcription polymerase chain reaction
SELEX: Systematic evolution of ligands by exponential enrichment
SPR: Surface plasmon resonance
TPFI: Tissue factor pathway inhibitor
VEGF: Vascular endothelial growth factor
XNA: Xeno-nucleic acids
Z: 6-Amino-5-nitro-2(1*H*)-pyridone
